# Dr. Allen Gideon Tripp

**Published:** 1912-08

**Authors:** 


Dr. Allen Gideon Tripp, Syracuse, 1900, a resident of Cicero, N. Y., died at the Hospital of the Good Shepard, Syracuse, June 28, aged 43.

